# Fungal Laccases and Fumonisin Decontamination in Co-Products of Bioethanol from Maize

**DOI:** 10.3390/toxins16080350

**Published:** 2024-08-10

**Authors:** Marianela Bossa, Noelia Edith Monesterolo, María del Pilar Monge, Paloma Rhein, Sofía Noemí Chulze, María Silvina Alaniz-Zanon, María Laura Chiotta

**Affiliations:** 1 Instituto de Investigación en Micología y Micotoxicología (IMICO), CONICET-Universidad Nacional de Río Cuarto (UNRC), Ruta Nacional 36 Km 601, Río Cuarto 5800, Argentina; mbossa@exa.unrc.edu.ar (M.B.); mmonge@exa.unrc.edu.ar (M.d.P.M.); schulze@exa.unrc.edu.ar (S.N.C.); 2 Instituto de Biotecnología Ambiental y de la Salud (INBIAS), Consejo Nacional de Investigaciones Científicas y Técnicas (CONICET)-Universidad Nacional de Río Cuarto (UNRC), Ruta Nacional 36 Km 601, Río Cuarto 5800, Argentina; nmonesterolo@exa.unrc.edu.ar; 3 Facultad de Ciencias Exactas, Físico-Químicas y Naturales, Universidad Nacional de Río Cuarto (UNRC), Ruta Nacional 36 Km 601, Río Cuarto 5800, Argentina; palomarhein6@gmail.com

**Keywords:** fungal laccases, fumonisin B_1_, maize steep liquor, decontamination, food safety

## Abstract

Maize (*Zea mays* L.) may be infected by *Fusarium verticillioides* and *F. proliferatum*, and consequently contaminated with fumonisins (FBs), as well as the co-products of bioethanol intended for animal feed. Laccase enzymes have a wide industrial application such as mycotoxin degradation. The aims were to isolate and identify fungal laccase-producing strains, to evaluate laccase production, to determine the enzymatic stability under fermentation conditions, and to analyse the effectiveness in vitro of enzymatic extracts (EEs) containing laccases in degrading FB_1_. Strains belonging to *Funalia trogii*, *Phellinus tuberculosus*, *Pleurotus ostreatus*, *Pycnoporus sanguineus* and *Trametes gallica* species showed laccase activity. Different isoforms of laccases were detected depending on the evaluated species. For the FB_1_ decontamination assays, four enzymatic activities (5, 10, 15 and 20 U/mL) were tested, in the absence and presence of vanillic acid (VA) and 2,2,6,6-tetramethylpiperidine-N-oxyl (TEMPO) as redox mediators (1 and 10 mM). *Trametes gallica* B4-IMICO-RC EE was the most effective strain in buffer, achieving a 60% of FB_1_ reduction. Laccases included in EEs remained stable at different alcoholic degrees in maize steep liquor (MSL), but no significant FB_1_ reduction was observed under the conditions evaluated using MSL. This study demonstrate that although laccases could be good candidates for the development of a strategy to reduce FB_1_, further studies are necessary to optimise this process in MSL.

## 1. Introduction

Maize is a relevant crop for the economy of several countries, including Argentina, due to its production and exportation volumes. In this country, the grains are an important source for bioethanol production, with the resulting co-products being utilized both as direct and in the manufacture of animal feeds [1,2]. Several studies have demonstrated that mycotoxins (toxic fungal secondary metabolites that often contaminate food and feed), are resistant to the bioethanol production process from maize. In fact, the mycotoxins can concentrate up to three times [3].

Fungal species producers of mycotoxins can infect crops at pre- and post-harvest stages [4]. In regard to toxicity and food safety, fumonisins (FBs) are considered ones of the most important mycotoxins, which are produced by *Fusarium verticillioides* and *F. proliferatum*, members of the *Fusarium fujikuroi* species complex, and *Aspergillus* section *Nigri*. These species are implicated in maize ear rot and they are of particular concern due to their high potential to produce these toxins [5,6,7]. Fumonisins are mycotoxins linked to with leukoencephalomalacia in horses, liver and kidney toxicity in rodents, porcine pulmonary edema, and neural tube defects in humans [8]. Within the fumonisin type B group, with fumonisin B1 (FB_1_) is the most important in toxicity and occurrence terms [9]. Based on its toxicity, FB_1_ has been classified by the International Agency for Research on Cancer (IARC) as a group 2B carcinogen [10]. Animals are exposed to feed mycotoxin contamination, with their subsequent accumulation in tissues, milk, and eggs. In processed foods, these metabolites are not eliminated during the conventional industrial manufacturing [11].

Due to its harmful effect, it is essential to apply mycotoxin reduction or decontamination strategies in industrial processes [12]. Among the strategies that allow reducing contamination levels with FBs, biological methods based on the use of microorganisms or enzymes able to metabolise, destroy or deactivate toxins into stable and less toxic, even innocuous, compounds, have been implemented. The biological agents application and their enzymes in industrial processes is a specific and effective methodology and allows regarding most problems related to environmental sustainability, especially with respect to the dangerous chemicals use, with a minor impact on food quality [13,14]. To reduce the mycotoxins risk in bioethanol co-products and achieve greater acceptance of these substrates as feed ingredients, it is crucial to monitor contamination levels throughout the bioethanol production process and implement appropriate mitigation strategies [15]. In recent years, the mycotoxins enzymatic biodegradation in food and feed has attracted great interest [13,14,16,17,18,19,20,21,22], one of the most relevant enzymes for the development of this kind of strategies are laccases.

Laccases (benzenediol: oxygen oxidoreductases, EC 1.10.3.2) are enzymes classified within the multicopper oxidase group [23,24,25]. The laccases present two particular characteristics compared to other lignin-degrading enzymes (lignin peroxidase, versatile peroxidase, manganese peroxidase, aryl alcohol oxidase, decolorizing peroxidases and estereases): the use of O_2_ instead of H_2_O_2_ generating a more labile chemical environment, and produces water as a single by-product [24]. In this sense, these enzymes may be useful in many industrial applications, such as in the pulp and paper industry [26,27,28], textile industry [29,30,31,32], bioremediation [33,34,35,36,37,38,39], organic synthesis and food industry [23,40,41,42,43]. Laccases are widely synthesised by plants, insects, bacteria, and fungi, being those that belong to the Phylum Basidiomycota causing white rot, the most efficient and the most studied lignin degraders [24,44]. Different species are able to secrete isoenzymes (encoded by different genes) or isoforms (derived from the same gene but exhibiting different post-translational modifications) of laccases [13,45,46,47]. Their enzymatic stability is influenced by various factors, including temperature, pH, the glycosylation degree of the laccase molecule, protein conformation, hydrophobicity, the quantity of hydrogen bonds, the increase in the number of certain charged amino acids on the protein surface [13,48,49].

Fungal laccases are characterised by their high redox potential, which favors the oxidation of a wide variety of substrates, encompassing phenolic compounds, such as p-diphenols, o-diphenols, aminophenols and aryldiamines, inorganic ions and non-phenolic compounds [50]. The rate of substrate oxidation by laccase is mainly contingent upon the variation in redox potential between the substrate and the Cu T1 center at the enzyme’s active site, as on the chemical structure of the substrate [33]. To overcome these limitations of steric hindrance and high redox potential of the substrate, and optimise the oxidation efficiency by laccases, the use of effective redox mediators for laccases has been suggested [51,52].

Based on this background, in the present study the use of fungal laccases to decontaminate mycotoxins is proposed. The aims were to isolate and molecularly identify fungal laccase-producing strains; evaluate and compare the laccase production ability in submerged fermentation by the different strains; analyse the production of laccase isoenzymes by the different fungal strains; determine the enzymatic stability in the fermentation stage for the bioethanol production and; evaluate enzymatic extracts (EEs) containing laccases in the degradation of FB_1_ under in vitro assays in buffer and maize steep liquor (MSL), an intermediate by-product from maize generated during the bioethanol production process. This biotechnological tool would contribute with future applications in food safety.

## 2. Results

### 2.1. Production of Ligninolytic Enzyme by Basidiomycota

A total of 12 fruiting bodies were collected from decaying wood and isolated. From the analysis of enzyme ligninolytic production, it was observed that 8 strains, representing almost 67% of the total collected, were producers of these enzymes in both culture media at different incubation periods. The incubation periods in which the enzymes were detected varied among strains. No significant differences between both media were observed (*p* value > 0.01). The B3-IMICO-RC strain produced enzymes at the day 2 of incubation, whereas the strain B9-IMICO-RC, at day 12. The other strains showed production between 4 to 6 days. Those non producer strains of ligninolytic enzymes were not considered for further studies.

All ligninolytic enzyme producer strains were confirmed as laccase producers.

### 2.2. Molecular Identification of Laccase Producer Strains

The sequence data obtained from the amplified ITS and LSU regions allowed the identification of the 8 isolates producing laccases, with 100% homology. Four strains were identified at species level as *Trametes gallica* while the 2 strains as *Pycnoporus sanguineus*, 1 strain as *Phellinus tuberculosus* and 1 strain as *Pleurotus ostreatus*.

### 2.3. Specific Activity of Laccases

**Figure 1:**
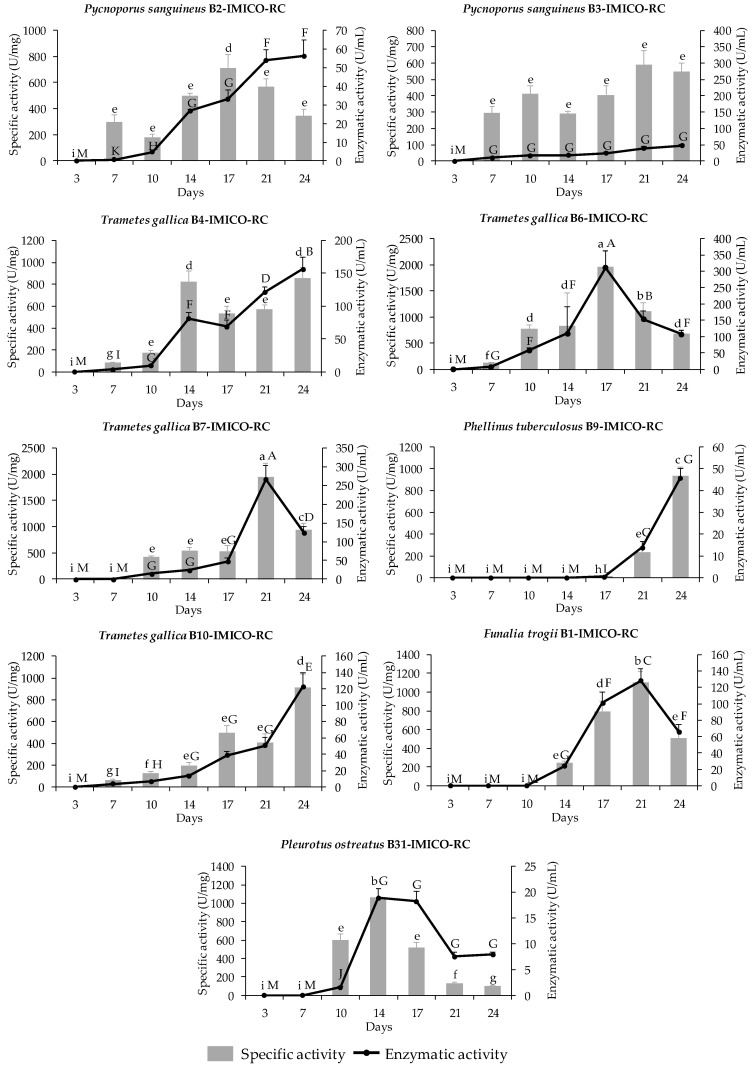
Specific and enzymatic activities of laccase enzymes from different enzymatic extracts of the laccase producing fungal isolates and the control strain (*Funalia trogii* B1-IMICO-RC), inoculated in broth for the laccase production. The enzymatic substrate used for the enzymatic activity determination was 2,2′-azino-di-3 ethylbenzothiazoline (ABTS). Error bars indicate the standard deviation within each treatment. Different capital letters denote significant differences between enzymatic activities (*p* value < 0.01), and different lowercase letters indicate significant differences between specific activities (*p* value < 0.01).

At 10 days of incubation, enzymatic activity was observed in the EEs of all strains, except *P. tuberculosus* B9-IMICO-RC and *Funalia trogii* B1-IMICO-RC strains (Figure 1). The *T. gallica* B6-IMICO-RC strain reached the maximum laccase activity (311.1 U/mL), followed by the *T. gallica* B7-IMICO-RC (266.7 U/mL), *T. gallica* B4-IMICO-RC (156.6 U/mL), *F. trogii* B1-IMICO-RC (128.2 U/mL) and *T. gallica* B10-IMICO-RC (122.7 U/mL) strains. The strains *P. sanguineus* B2-IMICO-RC, *P. sanguineus* B3-IMICO-RC and *P. tuberculosus* B9-IMICO-RC showed activities among 45.9 and 56.2 U/mL. *Pleurotus ostreatus* B31-IMICO-RC was the strain with the lowest laccase activity (18.9 U/mL).

**Figure 2:**
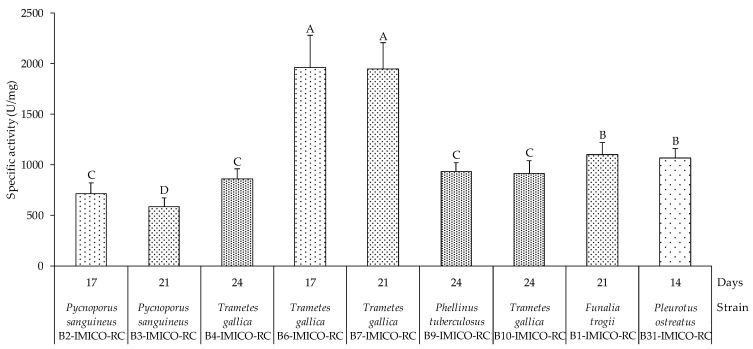
Maximum specific activity observed during the 24-day incubation period of the laccase enzymes contained in the different enzymatic extracts of the laccase producing fungal isolates and the control strain (*Funalia trogii* B1-IMICO-RC), inoculated in broth for the laccase production. The enzymatic substrate used for the enzymatic activity determination was ABTS. Error bars indicate the standard deviation within each treatment. Different letters indicate significant differences between specific activities (*p* value < 0.01).

The specific activity was detected after 7–10 days of incubation for all strains, except *F. trogii* B1-IMICO-RC and *P. tuberculosus* B9-IMICO-RC, which showed laccase specific activity at day 14 and 21, respectively (Figure 1). The highest specific activities were observed for the strains *T. gallica* B6-IMICO-RC (1957.6 U/mg) and *T. gallica* B7-IMICO-RC (1942.3 U/mg), followed by *F. trogii* B1-IMICO-RC (1097.7 U/mg) and *P. ostreatus* B31-IMICO-RC (1062.6 U/mg). The specific activities of the other laccases varied from 588 to 934.5 U/mg. Figure 2 shows the highest specific activities observed for each strain under evaluated conditions, within a 24-day incubation period.

**Figure 3:**
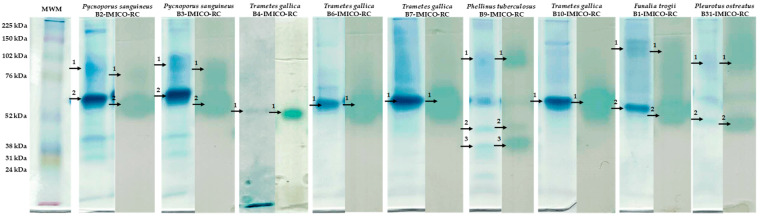
Native-PAGE (left side of each strain) vs. zymogram (right side of each strain) of the concentrated enzymatic extracts. Images of both gels were cut and presented one next to the other to show a better comparison of the results. Numbered arrows indicate the laccase or laccase isoenzyme synthesised by each strain. MW: molecular weight marker.

The results of the Native-PAGE and the zymogram are presented in Figure 3. The protein profile was variable among species. In relation to the strains belonging to the same species, *P. sanguineus* showed the same protein profile for both strains (B2-IMICO-RC and B3-IMICO-RC), whereas this behaviour was not observed for the strains of *T. gallica* (B4-IMICO-RC, B6-IMICO-RC, B7-IMICO-RC and B10-IMICO-RC).

As regards laccase profile, different isoenzymes were detected. *Pycnoporus sanguineus*, *P. ostreatus* and *F. trogii* showed two isoenzymes each, with apparent molecular weights of 80 and 60 kDa, 84 and 50 kDa, and 107 and 57 kDa, respectively. *Trametes gallica* strains produced a single laccase with an apparent molecular weight of approximately 60 kDa. *Phellinus tuberculosus* was the species with the higher number of laccase isoforms, with apparent molecular weights of 83, 51 and 38 kDa (Figure 3).

### 2.4. Fumonisin B_1_ Decontamination

**Figure 4:**
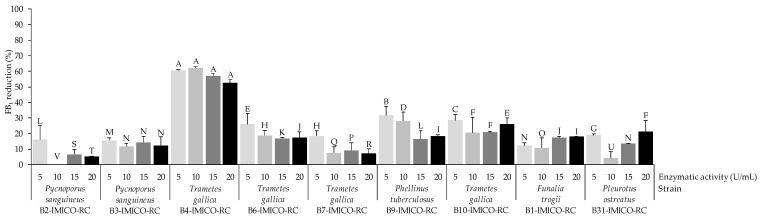
Reduction percentages of FB_1_ in the in vitro assays using buffer medium without redox mediators. A value of 100% signifies the total reduction of FB_1_ compared to the control (0% reduction). Error bars represent the standard deviation of the replicates for each treatment. Different letters on the bars denote significant differences between treatments (*p* value < 0.01).

Regarding the assay carried out in buffer, the results on the ability of EEs containing laccases to reduce FB_1_ in the absence of redox mediators are shown in Figure 4. In general, the enzymatic activity of 5 U/mL was more effective than the other evaluated activities. The EE from *T. gallica* B4-IMICO-RC exhibited the highest reduction level, with laccase activities ranging from 52.4% to 62.2% and no significant differences observed (*p* value > 0.01). Other efficient EEs in the reduction of FB_1_ were those from strains *P. tuberculosus* B9-IMICO-RC and *T. gallica* B10-IMICO-RC, reaching reduction percentages of 31.8 and 28.5%, respectively at 5 U/mL. Variable effectiveness was observed for the other EEs depending on the laccase activity and the strains (0–31.2%).

The addition of redox mediators was less efficient in degrading FB_1_ than the assays performed in the absence of them (Table 1). Evenmore, in most of the treatments there was no reduction of this mycotoxin. In the assay with 1 mM vanillic acid (VA), the highest percentage of FB_1_ decontamination (50.9%) was obtained by the EE of the *T. gallica* B4-IMICO-RC strain at the enzymatic activity of 5 U/mL. When 10 mM VA was used, only the EEs of *T. gallica* B6-IMICO-RC (20 U/mL) and *P. tuberculosus* B9-IMICO-RC (5 U/mL) reduced FB_1_ in low percentages (15.5 and 5.9%, respectively).

As regards the assay with 1 mM 2,2,6,6-tetramethylpiperidine-N-oxyl (TEMPO) no reduction of FB_1_ in any of the treatments was observed. Thus, these results were not included in Table 1. On the contrary, at 10 mM some EEs showed reductions, being the EE from *F. trogii* B1-IMCO-RC the most efficient (40.8%).

These results show that, in general, when the addition of one of the redox mediators reduced FB_1_, the other mediator did not have the same effect.

**Figure 5:**
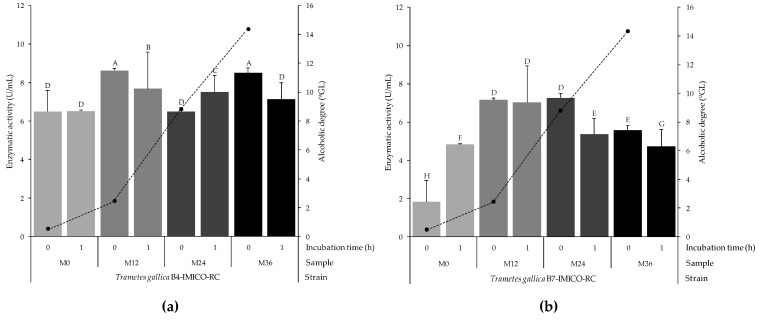
Stability of laccases from *Trametes gallica* B4-IMICO-RC (**a**) and *T. gallica* B7-IMICO-RC (**b**) under different alcoholic degrees in maize steep liquor (MSL) which was obtained from a bioethanol production process. M0, M12, M24 and M36 are samples collected at different times from the fermentation stage, corresponding to 0, 12, 24 and 36 h, respectively. The bars indicate the enzymatic activity (time 0 and 1 h) and the dotts linked by a dotted line represent the alcoholic degree of the MSL samples, measured at 0 h. Error bars indicate the standard deviation within each treatment. Different letters show significant differences between treatments (*p* value < 0.01).

A stability assay was performed to evaluate whether the alcoholic grade, the pH and any other factor of the MSL could affect the laccase activity (Figure 5). No laccase activity was detected in the MSL collected from the bioethanol industry. The initial pH of the MSL (M0) was 4.9 and varied to around 4.4 in the samples collected at 12 h (M12) from the beginning of the fermentation, remaining constant in the other samples (M24 and M36). On the contrary, the alcoholic level increased from 0.51 to 14.34 °GL. The laccase activity of the EEs from the *T. gallica* B4-IMICO-RC strain remained constant at M0, while *T. gallica* B7-IMICO-RC an increase was observed. When the alcoholic degree was higher (M12 to M36), the laccase activity slightly decreased. In general, the lacasses present in the EEs showed stability at different alcoholic degrees after 1 h of incubation, since the lacasse activity ranged from 4.8 to 7.7 U/mL.

The results of the in vitro decontamination assay in MSL showed that no significant FB_1_ reduction was detected when EEs containing laccases from *T. gallica* B4-IMICO-RC and B7-IMICO-RC were added.

## 3. Discussion

In the present study, the Phylum Basidiomycota species producers of white rot were isolated and studied due to its high ability to produce laccase enzymes [53]. Through the analysis of ligninolytic enzyme production, it was determined that several isolates were able to synthesise enzymes at different incubation times, depending on each isolate and on the physiology of the fungus [54]. Molecular identification determined that the ligninolytic isolates under study belong to the species *P. tuberculosus*, *T. gallica*, *F. trogii*, *P. sanguineus* and *P. ostreatus*. Previously, several authors reported strains within most of these species, that were producers of laccase enzymes [55,56,57,58,59,60,61,62,63]. The results observed in the present study agree with these researches, since the laccase activity of the evaluated strains was confirmed for all of them by the assay using ABTS. This is the first report that reveals laccase production by a strain of *Phellinus tuberculosus*.

Although at 10 days of incubation all strains showed enzymatic activity, the production levels were lower in comparison with longer incubation times, showing *T. gallica* B6-IMICO-RC strain the highest yield at 17 days followed by B7-IMICO-RC strain at 21 days. Additionally, both strains were the most efficient, showing the highest specific activities. Laccases have been largely used in industry processes, being the production times a crucial factor [26]. Unlike bacterial laccases, fungal laccases have a higher redox potential, but one of their limitations is the extended period of time they need to be synthesised. In the industrial fermentation processes, high laccase levels are required at short production times, thus different alternatives are necessary to achieve these conditions. The laccases expression in hosts that are easily cultivated and manipulated can generate high production of laccases in a short time and reduce costs [53,64].

In general, molecular weights of fungal laccases vary between 50 and 140 kDa [65]. In the present work, the results of the zymogram were compared with Native-PAGE to know the apparent molecular weight of the laccases and the production of laccase isoenzymes contained in the EEs of the different strains. Our results show that the production of laccase isoenzymes was variable among species but not within the same fungal species. Besides the species, other factors that influence the synthesis and secretion of laccase isoenzymes are the growth phase of the fungus, culture conditions, the addition of inducers to the culture medium and the conditions of the production process [46,47,66]. It has been reported that the isoenzymes proportion depends on the kind of substrate employed and this proportion plays an important role in the degradation process [67]. Moreover, the fungal strains producers of more than one laccase isoenzyme would be useful to improve the efficiency of some substrates degradation [68]. Since different isoenzymes have diverse multimeric structures, those chemical characteristics would confer these oxidation advantages [69]. Despite this, the selected EEs in the present work as potential FBs degraders, belonging to *Trametes gallica*, showed only one isoenzyme in the laccase profile with high efficiency.

Laccase enzymes are extensively employed as biotechnological tools due to their versatility as biocatalysts [70]. They are widely utilised in the food, textile, and pulp and paper industries, besides to be used in detoxification process of several compounds including mycotoxins such as AFB_1_ [16,17,71,72,73,74], FB_1_ [18], zearalenone [18,22,75], and ochratoxin A [18]. Nevertheless, the enzyme applications for mycotoxin detoxification in the food and feed industry are currently limited [76]. At present study, in vitro assays for the decontamination of FB_1_ were conducted. In the buffer assay, all the EEs containing laccases showed degradation of FB_1_ with different reduction percentages. The efficiency of *T. gallica* B4-IMICO-RC strain is highlighted in comparison to the EEs from the other strains, being 50.2% more efficient than *P. tuberculosus* B9-IMICO-RC strain, which follows in order of importance for FB_1_ decontamination. These EEs reached higher FB_1_ reduction percentages in the absence of redox mediators. Under similar conditions, other authors reported no direct degradation of FB_1_ by a purified laccase from *Pleurotus eryngii* [18]. To date, there are no commercial biotechnological inputs based on laccase enzymes for FB_1_ decontamination. Nevertheless, a commercial esterase FumD (EC 3.1.1.87) (FUMzyme^®^; BIOMIN, Tulln, Austria) has already been used for the reduction of this mycotoxin using contaminated maize, reaching high reduction percentages [77].

An efficient redox mediator must function as a perfect substrate for an enzyme with a high turnover rate, should produce free radicals to facilitate the oxidation of the target compound away from the active site, and should deliver efficient results [78]. Regarding laccases, numerous studies have demonstrated that redox mediators, readily oxidised by these enzymes, increase mycotoxin degradation by facilitating the oxidation of the substrate outside the enzyme’s active site [79]. Loi et al. [18] evaluated the degradation of FB_1_ with different redox mediators, such as acetosyringone, syringaldehyde, p-coumaric acid, ferulic acid, 1-hydroxybenzotriazole, TEMPO, ABTS and phenol red, and determined that TEMPO was the most efficient in FB_1_ degradation. Contrarily, under the conditions of the present study, the use of VA and TEMPO did not improve the oxidation of FB_1_ when compared with the treatments without redox mediators. This is the first study of the use of VA as a redox mediator in the FB_1_ degradation by laccase enzymes. Although no enhancement of the laccases effectivity was detected in the present work, and considering that VA is a natural compound with multiple benefits in the food and feed industry [80,81,82,83], more evaluations will be performed in order to optimise the degradation process in the presence of VA and to determine whether this redox mediator could increase in any condition the FB_1_ degradation.

In previous studies, the efficiency in AF decontamination by EEs from the same strains included in the present work was demonstrated under in vitro assays, and laccase performance was improved in the presence of VA [16]. However, in this study, the highest FB_1_ degradation levels were observed in the absence of redox mediators. These results also differ from the evaluation of AFs decontamination in the laccase activities that were necessary to degrade the mycotoxins, being necessary lower activities to degrade FB_1_ than to degrade AFB_1_ in the absence of redox mediators. No other reports have been found in relation to this data. These results could contribute to the industrial processes that require laccases, since lower investments in the acquisition of laccases and no costs for redox mediators are necessary for FB_1_ degradation.

Regarding the enzymatic stability assay in MSL at different alcoholic degrees, the results indicate that the laccases remained active under the evaluated conditions. Although in most cases the enzymatic activity slightly decreases, an increase was observed in some cases (Figure 5). Comparable results have been demonstrated by Zucca et al. [84], who evaluated the laccase stability with ethanol addition, and found that at an alcohol degree of 10 °GL, residual enzymatic stability remained stable. In addition, other authors determined that ethanol can be used as an inducer to overproduce laccases, even in a more efficient way than other compounds [70,85,86,87]. At the same time, laccases increase the ethanol production in simultaneous saccharification and fermentation by favouring the delignification [79].

Considering that the conditions previously evaluated, which simulate the real conditions of the fermentation process, do not affect the activity of the laccase enzymes contained in the EEs, the study of FB_1_ degradation in MSL was conducted. However, the data showed that no FB_1_ reduction was observed under the present experimental conditions. Although these results were not ideal, this is the first attempt to decontaminate FB_1_ in a bioethanol co-product from maize in order to obtain safe products intended for animal feed. Currently, we are evaluating different conditions such as the amounts of the EEs containing laccases and the use of redox mediators, in order to achieve relevant reductions. The optimization of these parameters, in addition to additional information from previous studies [16] could be useful to develop an efficient strategy to decontaminate FB_1_ and AFB_1_ simultaneously, both important mycotoxins in maize and sub-products.

## 4. Conclusions

In the present work 8 fungal laccase-producing strains were isolated and identified. Strains belonging to *Trametes gallica* species showed the highest specific and enzymatic activities during periods of time that could be useful for industrial applications. According to the studied laccases, different isoenzymes were detected among diverse species, but not among strains within a same species. Regarding the results observed in the present study, the use of VA and TEMPO as natural and synthetic redox mediators, respectively, would not be necessary to enhance the FB_1_ decontamination by laccases included in enzymatic extracts under assays performed in buffer. This is the first report on the evaluation of VA as redox mediator for laccase-mediated decontamination of FB_1_. The laccases produced by *Trametes gallica* were stable in MSL with different alcoholic degrees. In this sense, a strategy based on the use of these laccases could be developed to be applied during the fermentation stage. Since the laccases evaluated in this work did not reduce FB_1_ when applied as part of an EEs, alternative strategies involving these enzymes could be explored in future research.

In conclusion this research provides information about fungal laccases with potential industrial application. Development and implementation of strategies using this enzymes could contribute to food safety, reducing the risk of FB_1_ contamination in maize by-products. The present work proposes the EEs from *Trametes gallica* B4-IMICO-RC strain as good candidate for these biotechnological tools.

## 5. Materials and Methods

### 5.1. Fungal Collection and Isolation

A total of 12 fruiting bodies within the Basidiomycota phylum were collected from decaying wood located in the Universidad Nacional de Río Cuarto (UNRC) (33°06′52.0″ S 64°18′06.0″ W). They were washed 3 times with sterile distilled water and superficially disinfected using 70% ethanol for 30 s, and 0.5% sodium hypochlorite for 1 min, repeating each step 3 times. Disinfection with 70% ethanol was repeated one more time, and finally the fruiting bodies were rinsed with sterile distilled water and dried on sterile filter paper [88]. Thin slices of the fruiting body were cultured in Potato Dextrose Agar (PDA) supplemented with 0.01% chloramphenicol to obtain axenic cultures. The incubation was performed in darkness at 30 ± 1 °C for up to 15 days. A commercial *Pleurotus* sp. strain was also included within the pool of strains under evaluation.

The strains were preserved in 20% glycerol at −80 °C and incorporated into the culture collection of the Instituto de Investigación en Micología y Micotoxicología (IMICO), CONICET-UNRC.

### 5.2. Analysis of Ligninolytic Enzyme Production

The isolated fungi were evaluated to determine their ligninolytic activity by degradation of bromophenol blue, used as an indicator substrate [89]. The isolates were cultured in PDA and Malt Extract Agar (MEA), both supplemented with bromophenol blue (0.2 g/L). Plates were incubated at 30 ± 1 °C in darkness during 21 days. The strain of *Funalia trogii* B1-IMICO-RC was included as a positive control. The discoloration of the culture media indicated the presence of ligninolytic enzymes [88,89,90], and producing strains were selected for the following assays.

### 5.3. Enzymatic Production and Laccase Activity Determination

Laccase production by the strains producers of ligninolytic enzymes was evaluated. Enzymatic extracts (EEs) containing laccases were obtained according to Bossa et al. [16]. Laccase enzymatic activity was measured by spectrophotometry at a wavelength of 420 nm (UV-VISIBLE Rayleigh LUV200B), using ABTS (Sigma-Aldrich, St. Louis, MO, USA) as an enzyme substrate. For the laccase activity determination, 0.1 M sodium acetate buffer (pH 3.6), 0.5 mM of ABTS and a standardized volume of EE were used, in a final volume of 1 mL. One unit (U) of enzyme activity was determined as the quantity of enzyme necessary to oxidize 1 µmol of ABTS per minute at 25 ± 1 °C. The blanks were realised with sterile broth.

Laccase activity was calculated using the formula described by Baltierra-Trejo et al. [91]:

(1) $$U/\mathrm{mL}=\frac{∆A/∆\min\;\times \;V}{v\;\times \;\varepsilon \;\times \;d}$$

where ∆A is the absorbance change per minute at 420 nm, V is the total reaction volume (mL), v is the enzymatic extract volume (mL), ε is the extinction coefficient of ABTS at 420 nm (ε_420_ = 3.6·10^4^/M·cm) and d is the light path of cuvette (cm).

#### 5.3.1. Laccase Enzymatic and Specific Activities

The enzymatic (as described above) and specific activities of the EEs were evaluated at 3, 7, 10, 14, 17, 21 and 24 incubation days. The relationship between the enzymatic activity and the total protein concentration present in the sample was defined as the specific activity (U/mg). The laccase specific activity for each EE was calculated using the following equation:

(2) $$\mathrm{Specific}\;\mathrm{activity}\;(U/\mathrm{mg})=\frac{\mathrm{Enzymatic}\;\mathrm{activity}\;(U/\mathrm{mL})}{\mathrm{Protein}\;\mathrm{concentration}\;(\mathrm{mg}/\mathrm{mL})}$$

Bradford’s method was employed to determine the total protein concentration in the EEs, using bovine serum albumin (Sigma-Aldrich, St. Louis, MO, USA) as standard [92].

The *F. trogii* B1-IMICO-RC strain, previously used as positive control, was included in these studies within the pool of strains under evaluation.

#### 5.3.2. Electrophoresis in Polyacrylamide Gels

In order to characterise the protein pattern and to detect the enzymatic activity of the EEs, a Native-PAGE was performed. For this, 7 mL of the EEs were lyophilized and resuspended in 500 µL of sterile broth employed for laccase production [16]. The total protein concentration was measured and according to this data, protein solutions were standardised at 28 µg/µL and 0.25 µg/µL of total protein for Native-PAGE and zymogram, respectively. Samples were resuspended in Laemmli [93] sample buffer and plated in equivalent amounts for all EEs.

The native gel was prepared according to the methodology of Arndt et al. [94], with modifications. Enzymatic extracts were separated on 12% polyacrylamide gels. The apparent molecular weight of the laccases was calculated by comparison with the relative mobility of the molecular weight marker (Amershan^TM^-ECL^TM^ Rainbow^TM^ Marker-Full Range, GE Healthcare Life Sciences, Piscataway, NJ, USA), with bands ranging between 12–225 kDa.

After the electrophoretic run and to determine the protein profile, the protein bands were visualised by incubating the gel with a Coomassie Blue-R250 solution (Coomassie R-250, Sigma-Aldrich, St. Louis, MI, USA), acetic acid and methanol (0.25 *w*/*v*: 45 *v*/*v*: 10% *v*/*v*, by volume of water), for 24 h in gentle shaking. After this period, the gel was bleached with a solution composed of 40% methanol, 10% acetic acid and 50% distilled water.

To obtain a laccase isoenzyme zymogram, a native gel was also carried out. After the electrophoretic run, the gel was washed with distilled water 4 times for 15 min at room temperature while shaking (150 rpm). Subsequently, the gel was incubated with an ABTS solution (0.1 M sodium acetate buffer (pH 3.6) and 0.5 mM ABTS), and the enzymatic activity was determined within 6 min, by the colouring of the bands corresponding to laccases. Bovine serum albumin was used as a negative control.

### 5.4. Molecular Identification of the Fungal Species

#### 5.4.1. DNA Extraction

Each laccase-producing strain was inoculated in 75 mL of Wickerham broth [95] and incubated at 28 ± 1 °C under shaking (150 rpm) for 7 to 25 days. The mycelium was recovered by filtration through non-gauze milk filters (Ken AG, Ashland, OH, USA), washing it with sterile distilled water and drying it between clean paper towels. The dried mycelia were stored at −20 °C until grounding with liquid nitrogen. DNA extraction was performed by cetyltrimethylammonium bromide (CTAB) method [9]. Genomic DNA was quantified using a spectrophotometer (model ND-1000; NanoDrop Technologies Inc., Wilmington, DE, USA) and the quality was confirmed by electrophoresis in 1X TAE buffer (Tris-acetate-EDTA, 40 mM Tris-base (pH 8), 0.5 mM EDTA, glacial acetic acid) on 0.8% agarose gels stained with Ethidium Bromide (5%) for 30 min at 70 V using a Mini-Sub^®^ Cell GT (Bio-Rad Laboratories, Hong Kong, China). Power was supplied by Power PAC 300 (Bio-Rad Laboratories, Hercules, CA, USA).

#### 5.4.2. PCR Amplification and Sequencing

The strains were identified by the amplification of the ITS (Internal Transcribed Spacers) region (approx. 600–700 bp) of the ARNr using the primer pair ITS-1F/ITS4 (5′-TCCGTAGGTGAACCTGCGG-3′/5′-TCCTCCGCTTATTGATATGC-3′ [96]), and the D1-D2 region of the 28S gene (LSU, large subunit of the ARNr), using the primer pair NL1/NL4 (5′-GCATATCAATAAGCGGAGGAAAAG-3′/5′-GGTCCGTGTTTCAAGACGG-3′ [97]).

Amplifications were performed in a PCR cycler (Biometra Tone Series, Analytik Jena GmbH, Jena, Germany). For ITS and LSU regions, the annealing temperatures were 58 °C and 51 °C, respectively. PCR products were separated by electrophoresis in the same conditions as previously described. A 100 bp DNA ladder (New England Biolabs, Ipswich, MA, USA) as a size standar was included. The PCR products were purified and sequenced by Macrogen Inc., (Seoul, Republic of Korea). The sequences were aligned using the BioEdit Sequence Alignment Editor 7.1.3.0 [98], and compared with reference sequences available on GenBank (National Center for Biotechnology Information, NCBI) databases for identification of the fungal species.

### 5.5. Fumonisin Decontamination by Enzymatic Extracts Containing Laccases

#### 5.5.1. In Vitro Assay in Buffer Medium

The EEs with laccase activity were tested for FB_1_ degradation according to Loi et al. [18], with some modifications. Different EEs dilutions from each strain were prepared to obtain final laccase activities of 5, 10, 15 and 20 U/mL. A standard solution of FB_1_ (Sigma-Aldrich, St. Louis, MO, USA) was added to the reaction mixtures (final concentration 1 μg/mL). This experiment was conducted using sodium acetate buffer (1 mM, pH 5) at a final volume of 500 µL. For negative controls, the EEs were replaced with an equal volume of sterile broth. This assay was performed in the absence and in the presence of TEMPO and VA as redox mediators at two different concentrations (1 mM and 10 mM) in independent assays. The reactions were incubated at 30 ± 1 °C for 3 days in the dark. All the assays were performed in triplicate. After the incubation period, the reactions were stopped in boiling water for 5 min.

The detection of FB_1_ degradation in the different treatments was carried out by high-performance liquid chromatography-tandem mass spectrometry (HPLC-MS/MS) according to Cendoya et al. [99]. A Waters Alliance 2695 HPLC instrument (Waters Corporation, Milford, MA, USA) equipped with a Waters 2695 diode array detector connected to a tandem quadrupole (triple) analyzer mass spectrometer (Micromass^®^-Quattro Ultima™ Platinum, Waters-Micromass Ltd, Manchester, UK) was used with an electrospray ionization (ESI) source. Chromatographic separation was performed on an XBridge™ C18 column (3.5 μm, 2.1 × 150 mm) coupled to a XBridge BEH C18 Sentry Guard Cartridge pre-column (159 Å, 3.5 μm, 2.1 × 10 mm), all from Waters (Milford, MA, USA). The mobile phase consisted of a gradient of 1% aqueous formic acid and 1% formic acid in methanol, at a flow rate of 0.2 mL/min. The column temperature was maintained at 20 °C. The nitrogen flow was adjusted to 109 and 726 L/h for cone and desolvation gases, respectively. The interface was worked in positive ionization mode. The N_2_ was used as nebulization and desolvation gas at 150 and 200 °C, respectively. The capillary voltage was 3.00 kV. The determination of FB_1_ was performed using the multiple reaction monitoring (MRM) method. The precursor ion of FB_1_ [M^+^H]^+^, the two product or daughter ions (trace *m*/*z* 722 > 35) and the retention time were monitored for qualitative and quantitative analyses. Data acquisition and processing were performed using Mass Lynx V.4.1 software (Copyright 2005, Waters Corporation, Milford, MA, USA). Pure FB_1_ solutions were used as external standards (Sigma-Aldrich, St. Louis, MO, USA). The detection limit (LOD) and quantification limit (LOQ) was 0.01 and 0.05 ng/mL, respectively.

#### 5.5.2. In Vitro Assay in Maize Steep Liquor

##### Enzymatic Stability

The most efficient EE in degrading FB_1_ was selected. Additionally, the EE from *Trametes gallica* B7-IMICO-RC, whose effectiveness for AFB_1_ degradation was demonstrated in previous studies [16], was also included in this assay. Different samples of MSL were collected from a bioethanol production industry during fermentation at 0, 12, 24 and 36 h, with alcohol concentrations of 0.51, 2.45, 8.8 and 14.34 °GL, respectively. The pH was determined (Model 250A, Orion Research, Inc., Boston, MA, USA) for each sample, being 4.92, 4.39, 4.38 and 4.39, respectively. For this assay, the EEs of *T. gallica* B4-IMICO-RC and B7-IMICO-RC were added to MSL samples (10%, *v*/*v*) at enzymatic activities of 28 U/mL and 15 U/mL, respectively, and incubated at 33 ± 1 °C under static conditions in the dark. The reactions were carried out in triplicate and negative controls were included. The reaction mixtures were centrifuged at 10,000 rpm for 5 min (Sorvall ST 16, Thermo Fisher Scientific, Waltham, MA, USA). The supernatant was collected to determine laccase activity at 0 and 1 h.

##### Decontamination Assay

The substrate used in this assay was the MSL collected at 0 h, as previously described. Natural incidence of FB_1_ was determined to know the initial contamination level of the sample and add to each reaction mixture the necessary amounts of a standard solution of FB_1_ to obtain final concentrations of 5 ppm. Previously, the sample was dried and homogenized, as it was described in Bossa et al. [16]. Fumonisin extraction was carried out using a Quick, Easy, Cheap, Effective, Rugged, and Safe extraction procedure (QuEChERS), in accordance with Dzuman et al. [100]. Fumonisin B_1_ was detected and quantified via HPLC-MS/MS.

Different volumes of the EEs were combined with the MSL to achieve laccase activities of 5 and 20 U/mL, without using redox mediators. Negative controls were included. The assay was performed at 33 ± 1 °C for 60 h with a 95% occupancy rate to replicate the fermentation conditions. Each reaction was carried out in triplicate. To stop the reactions, they were exposed to boiling water for 5 min. The residual FB_1_ was assessed as detailed in Section 5.5.1.

### 5.6. Statistical Analyses

The statistical analyses were performed using InfoStat version 2020p software [101]. The data were presented as mean ± standard error. Data on specific and enzymatic activity were analysed by nonparametric Kruskal–Wallis test followed by Bonferroni’s post hoc comparison method at a probability level of *p* value < 0.01. Data on stability enzymatic and FB_1_ reduction percentages in buffer and in MSL were subjected to analysis of variance (ANOVA) followed by Fisher’s test (LSD) to determine the significant differences of each of the analyses (*p* value < 0.01).

## Figures and Tables

**Table 1:** Reduction percentages of FB_1_ in the presence of redox mediator in sodium acetate buffer.

		FB_1_ Reduction (%) *		
Strain	Enzymatic Activity (U/mL)	Vanillic Acid (1 mM)	Vanillic Acid (10 mM)	TEMPO (10 mM)
*Pycnoporus sanguineus* B2-IMICO-RC	10	4.5 ± 6.3 ^J^	0	0
	20	0	0	5.2 ± 7.4 ^I^
*Pycnoporus sanguineus* B3-IMICO-RC	10	6.6 ± 9.3 ^I^	0	0
*Trametes gallica* B4-IMICO-RC	5	50.9 ± 0.5 ^A^	0	0
	10	34.4 ± 15.1 ^C^	0	0
	15	16.16 ± 22.9 ^E^	0	0
*Trametes gallica* B6-IMICO-RC	15	15.6 ± 21.0 ^F^	0	0
	20	0	15.5 ± 22.0 ^F^	0
*Trametes gallica* B7-IMICO-RC	5	4.8 ± 9.7 ^I^	0	19.6 ± 10.4 ^D^
	20	0	0	4.2 ± 5.9 ^J^
*Phellinus tuberculosus* B9-IMICO-RC	5	1.4 ± 2.9 ^K^	5.9 ± 8.2 ^I^	0
	10	12.8 ± 4.3 ^G^	0	16.5 ± 9.8 ^E^
	15	3.7 ± 5.2 ^K^	0	3.4 ± 4.8 ^K^
	20	2.7 ± 1.1 ^K^	0	0
*Trametes gallica* B10-IMICO-RC	10	9.8 ± 13.9 ^H^	0	0
	15	11.4 ± 1.2 ^H^	0	0
*Funalia trogii* B1-IMICO-RC	5	0	0	8.8 ± 5.7 ^H^
	10	0	0	31.5 ± 1.4 ^C^
	15	0	0	37.1 ± 11.0 ^C^
	20	2.3 ± 3.3 ^K^	0	40.8 ± 12.7 ^B^
*Pleurotus ostreatus* B31-IMICO-RC	20	0	0	3.4 ± 4.8 ^K^

* Data are presented as mean ± standard deviation. Different letters indicate significant differences between treatments (*p* value < 0.01). The missing results for some enzymatic activities as well as the assay with 1 mM TEMPO, were excluded from the table because these treatments did not reduce FB_1_.

## Data Availability

Data is contained within the article.

## References

[1] Bolsa de Comercio de Rosario (BCR) Maíz: Balance de Oferta y Demanda en Argentina. https://www.bcr.com.ar/es/mercados/investigacion-y-desarrollo/informativo-semanal/estadisticas-informativo-semanal/maiz-23.

[2] Ministerio de Agricultura, Ganadería y Pesca de la Argentina (MAGyP) (2023). Estimaciones agrícolas. https://www.magyp.gob.ar/sitio/areas/estimaciones/_archivos/estimaciones/230000_2023/230800_Agosto/230817_Informe%20Mensual%20al%2017_08_2023.pdf.

[3] Buenavista R.M.E., Siliveru K., Zheng Y. (2021). Utilization of Distiller’s Dried Grains with Solubles: A review. J. Agric. Food Res..

[4] Gruber-Dorninger C., Jenkins T., Schatzmayr G. (2019). Global mycotoxin occurrence in feed: A ten-year survey. Toxins.

[5] Dinolfo M.I., Martínez M., Castañares E., Arata A.F. (2022). *Fusarium* in maize during harvest and storage: A review of species involved, mycotoxins, and management strategies to reduce contamination. Eur. J. Plant Pathol..

[6] Logrieco A., Battilani P., Leggieri M.C., Jiang Y., Haesaert G., Lanubile A., Mahuku G., Mesterhazy A., Ortega-Beltran A., Pasti M. (2021). Perspectives on global mycotoxin issues and management from the mycokey maize working group. Plant Dis..

[7] Wigmann É.F., Meyer K., Cendoya E., Maul R., Vogel R.F., Niessen L. (2020). A Loop-Mediated Isothermal Amplification (LAMP) based assay for the rapid and sensitive group-specific detection of fumonisin producing *Fusarium* spp.. Int. J. Food Microbiol..

[8] Knutsen H.-K., Alexander J., Barregård L., Bignami M., Brüschweiler B., Ceccatelli S., Cottrill B., Dinovi M., Edler L., EFSA Panel on Contaminants in the Food Chain (CONTAM) (2017). Risks for animal health related to the presence of zearalenone and its modified forms in feed. EFSA J..

[9] Alaniz Zanon M.S., Clemente M.P., Chulze S.N. (2018). Characterization and competitive ability of non-aflatoxigenic *Aspergillus flavus* isolated from the maize agro-ecosystem in Argentina as potential aflatoxin biocontrol agents. Int. J. Food Microbiol..

[10] International Agency for Research on Cancer (IARC) (1993). Monographs on the evaluation of carcinogenetic risks to humans. Some Naturally Occurring Substances: Food Items and Constituents, Heterocyclic Aromatic Amines and Mycotoxins.

[11] Stracquadanio C., Luz C., La Spada F., Meca G., Cacciola S.O. (2021). Inhibition of mycotoxigenic fungi in different vegetable matrices by extracts of *Trichoderma* species. J. Fungi.

[12] Dzuman Z., Stranska-Zachariasova M., Vaclavikova M., Tomaniova M., Veprikova Z., Slavikova P., Hajslova J. (2016). Fate of free and conjugated mycotoxins within the production of Distiller’s Dried Grains with Solubles (DDGS). J. Agric. Food Chem..

[13] Loi M., Glazunova O., Fedorova T., Logrieco A.F., Mulè G. (2021). Fungal laccases: The forefront of enzymes for sustainability. J. Fungi.

[14] Loi M., Fanelli F., Liuzzi V.C., Logrieco A.F., Mulè G. (2017). Mycotoxin biotransformation by native and commercial enzymes: Present and future perspectives. Toxins.

[15] Munkvold G.P., Arias S., Taschl I., Gruber-Dorninger C., Serna-Saldivar S.O. (2019). Mycotoxins in corn: Occurrence, impacts, and management. Corn: Chemistry and Technology.

[16] Bossa M., Alaniz-Zanon M.S., Monesterolo N.E., del Monge M.P., Coria Y.M., Chulze S.N., Chiotta M.L. (2024). Aflatoxin decontamination in maize steep liquor obtained from bioethanol production using laccases from species within the basidiomycota phylum. Toxins.

[17] Loi M., Fanelli F., Zucca P., Liuzzi V.C., Quintieri L., Cimmarusti M.T., Monaci L., Haidukowski M., Logrieco A.F., Sanjust E. (2016). Aflatoxin B_1_ and M_1_ degradation by Lac2 from *Pleurotus pulmonarius* and redox mediators. Toxins.

[18] Loi M., Fanelli F., Cimmarusti M.T., Mirabelli V., Haidukowski M., Logrieco A.F., Caliandro R., Mule G. (2018). In vitro single and combined mycotoxins degradation by Ery4 laccase from *Pleurotus eryngii* and redox mediators. Food Control.

[19] Loi M., De Leonardis S., Ciasca B., Paciolla C., Mulè G., Haidukowski M. (2023). Aflatoxin B_1_ degradation by Ery4 laccase: From in vitro to contaminated corn. Toxins.

[20] Qin X., Xin Y., Su X., Wang X., Wang Y., Zhang J., Tu T., Yao B., Luo H., Huang H. (2021). Efficient degradation of zearalenone by dye-decolorizing peroxidase from *Streptomyces thermocarboxydus* combining catalytic properties of manganese peroxidase and laccase. Toxins.

[21] Qin X., Xin Y., Zou J., Su X., Wang X., Wang Y., Zhang J., Tu T., Yao B., Luo H. (2021). Efficient degradation of aflatoxin B_1_ and zearalenone by laccase-like multicopper oxidase from *Streptomyces thermocarboxydus* in the presence of mediators. Toxins.

[22] Wang X., Bai Y., Huang H., Tu T., Wang Y., Wang Y., Luo H., Yao B., Su X. (2019). Degradation of aflatoxin B_1_ and zearalenone by bacterial and fungal laccases in presence of structurally defined chemicals and complex natural mediators. Toxins.

[23] Aza P., Camarero S. (2023). Fungal laccases: Fundamentals, engineering and classification update. Biomolecules.

[24] Zhou M., Fakayode O.A., Ren M., Li H., Liang J., Yagoub A.E.G.A., Fan Z., Zhou C. (2023). Laccase-Catalyzed lignin depolymerization in deep eutectic solvents: Challenges and prospects. Bioresour. Bioprocess..

[25] Bhardwaj P., Kaur N., Selvaraj M., Ghramh H.A., Al-Shehri B.M., Singh G., Arya S.K., Bhatt K., Ghotekar S., Mani R. (2022). Laccase-Assisted degradation of emerging recalcitrant compounds—A review. Bioresour. Technol..

[26] Moreno A.D., Ibarra D., Eugenio M.E., Tomás-Pejó E. (2020). Laccases as versatile enzymes: From industrial uses to novel applications. J. Chem. Technol. Biotechnol..

[27] Upadhyay P., Shrivastava R., Agrawal P.K. (2016). Bioprospecting and biotechnological applications of fungal laccase. 3 Biotech.

[28] Valls C., Roncero M.B. (2009). Using both xylanase and laccase enzymes for pulp bleaching. Bioresour. Technol..

[29] Su J., Shim E., Noro J., Fu J., Wang Q., Kim H.R., Silva C., Cavaco-Paulo A. (2018). Conductive cotton by in situ laccase-polymerization of aniline. Polymers.

[30] Su J., Noro J., Silva S., Fu J., Wang Q., Ribeiro A., Silva C., Cavaco-Paulo A. (2019). Antimicrobial coating of textiles by laccase in situ polymerization of catechol and p-phenylenediamine. React. Funct. Polym..

[31] Pezzella C., Giacobbe S., Giacobelli V.G., Guarino L., Kylic S., Sener M., Sannia G., Piscitelli A. (2016). Green routes towards industrial textile dyeing: A laccase based approach. J. Mol. Catal. B Enzym..

[32] Pazarlioǧlu N.K., Sariişik M., Telefoncu A. (2005). Laccase: Production by *Trametes versicolor* and application to denim washing. Process Biochem..

[33] Sá H., Michelin M., Silvério S.C., de Polizeli M.L.T., Silva A.R., Pereira L., Tavares T., Silva B. (2024). *Pleurotus ostreatus* and *Lentinus sajor-caju* laccases for sulfamethoxazole biotransformation: Enzymatic degradation, toxicity and cost analysis. J. Water Process Eng..

[34] Janusz G., Skwarek E., Pawlik A. (2023). Potential of laccase as a tool for biodegradation of wastewater micropollutants. Water.

[35] Vaithyanathan V.K., Vaidyanathan V.K., Cabana H. (2022). Laccase-Driven transformation of high priority pesticides without redox mediators: Towards bioremediation of contaminated wastewaters. Front. Bioeng. Biotechnol..

[36] Vidal-Limon A., García Suárez P.C., Arellano-García E., Contreras O.E., Aguila S.A. (2018). Enhanced degradation of pesticide dichlorophen by laccase immobilized on nanoporous materials: A cytotoxic and molecular simulation investigation. Bioconjug. Chem..

[37] Yang J., Li W., Bun Ng T., Deng X., Lin J., Ye X. (2017). Laccases: Production, expression regulation, and applications in pharmaceutical biodegradation. Front. Microbiol..

[38] Zhao Y.C., Yi X.Y., Zhang M., Liu L., Ma W.J. (2010). Fundamental study of degradation of dichlorodiphenyltrichloroethane in soil by laccase from white rot fungi. Int. J. Environ. Sci. Technol..

[39] Cañas A.I., Alcalde M., Plou F., Martínez M.J., Martínez Á.T., Camarero S. (2007). transformation of polycyclic aromatic hydrocarbons by laccase is strongly enhanced by phenolic compounds present in soil. Environ. Sci. Technol..

[40] López-Pérez M., Aguirre-Garrido J.F., Herrera-Zúñiga L., García-Arellano H., Atta-ur-Rahman (2024). Chapter 8—Structure, expression regulation, and applications of fungal laccases, an interesting prospective in biotechnology. Studies in Natural Products Chemistry.

[41] Osma J.F., Toca-Herrera J.L., Rodríguez-Couto S. (2010). Uses of laccases in the food industry. Enzyme Res..

[42] Ribeiro D.S., Henrique S.M.B., Oliveira L.S., Macedo G.A., Fleuri L.F. (2010). Enzymes in juice processing: A review. Int. J. Food Sci. Technol..

[43] Minussi R.C., Pastore G.M., Durán N. (2002). Potential applications of laccase in the food industry. Trends Food. Sci. Technol..

[44] Rodríguez-Couto S., Yadav A., Mishra S., Singh S., Gupta A. (2019). Chapter 13—Fungal laccase: A versatile enzyme for biotechnological applications. Recent Advancement in White Biotechnology through Fungi.

[45] González-González P., Gómez-Manzo S., Tomasini A., Martínez y Pérez J.L., García Nieto E., Anaya-Hernández A., Ortiz Ortiz E., Castillo Rodríguez R.A., Marcial-Quino J., Montiel-González A.M. (2023). Laccase production from *Agrocybe pediades*: Purification and functional characterization of a consistent laccase isoenzyme in liquid culture. Microorganisms.

[46] Bertrand B., Martínez-Morales F., Trejo-Hernández M.R. (2017). Upgrading laccase production and biochemical properties: Strategies and challenges. Biotechnol. Prog..

[47] Bertrand B., Martinez-Morales F., Trejo-Hernández M.R. (2013). Fungal laccases: Induction and production. Rev. Mex. Ing. Chim..

[48] Malhotra M., Kumar Suman S. (2021). Laccase-Mediated delignification and detoxification of lignocellulosic biomass: Removing obstacles in energy generation. Environ. Sci. Pollut. Res..

[49] Bilal M., Asgher M., Parra-Saldivar R., Hu H., Wang W., Zhang X., Iqbal H.M.N. (2017). Immobilized ligninolytic enzymes: An innovative and environmental responsive technology to tackle dye-based industrial pollutants—A review. Sci. Total Environ..

[50] Agrawal K., Chaturvedi V., Verma P. (2018). Fungal laccase discovered but yet undiscovered. Bioresour. Bioprocess..

[51] Mani P., Kumar V.T.F., Keshavarz T., Sainathan Chandra T., Kyazze G. (2018). The role of natural laccase redox mediators in simultaneous dye decolorization and power production in microbial fuel cells. Energies.

[52] Ashe B., Nguyen L.N., Hai F.I., Lee D.J., van de Merwe J.P., Leusch F.D.L., Price W.E., Nghiem L.D. (2016). Impacts of redox-mediator type on trace organic contaminants degradation by laccase: Degradation efficiency, laccase stability and effluent toxicity. Int. Biodeterior. Biodegrad..

[53] Khatami S.H., Vakili O., Movahedpour A., Ghesmati Z., Ghasemi H., Taheri-Anganeh M. (2022). Laccase: Various types and applications. Biotechnol. Appl. Biochem..

[54] Ang T.N., Ngoh G.C., Chua A.S.M. (2011). A quantitative method for fungal ligninolytic enzyme screening studies. Asia-Pac. J. Chem. Eng..

[55] Singh A.K., Iqbal H.M.N., Cardullo N., Muccilli V., Fernández-Lucas J., Schmidt J.E., Jesionowski T., Bilal M. (2023). Structural insights, biocatalytic characteristics, and application prospects of lignin-modifying enzymes for sustainable biotechnology. Int. J. Biol. Macromol..

[56] Cen Q., Wu X., Cao L., Lu Y., Lu X., Chen J., Fu G., Liu Y., Ruan R. (2022). Green production of a yellow laccase by *Coriolopsis gallica* for phenolic pollutants removal. AMB Express.

[57] Liu X., Deng W., Yang Y. (2021). Characterization of a novel laccase Lac-Yang1 from white-rot fungus *Pleurotus ostreatus* strain Yang1 with a strong ability to degrade and detoxify chlorophenols. Molecules.

[58] Zhang Y., Wu Y., Yang X., Yang E., Xu H., Chen Y., Chagan I., Yan J. (2021). Alternative splicing of heat shock Transcription Factor 2 regulates expression of the laccase gene family in response to copper in *Trametes trogii*. Appl. Environ. Microbiol..

[59] Zimbardi A.L.R.L., Camargo P.F., Carli S., Neto S.A., Meleiro L.P., Rosa J.C., De Andrade A.R., Jorge J.A., Furriel R.P.M. (2016). A high redox potential laccase from *Pycnoporus sanguineus* RP15: Potential application for dye decolorization. Int. J. Mol. Sci..

[60] He J.B., Feng T., Zhang S., Dong Z.J., Li Z.H., Zhu H.J., Liu J.K. (2014). Seven new drimane-type sesquiterpenoids from cultures of fungus *Phellinus tuberculosus*. Nat. Prod. Bioprospect..

[61] Ramírez-Cavazos L.I., Junghanns C., Ornelas-Soto N., Cárdenas-Chávez D.L., Hernández-Luna C., Demarche P., Enaud E., García-Morales R., Agathos S.N., Parra R. (2014). Purification and characterization of two thermostable laccases from *Pycnoporus sanguineus* and potential role in degradation of endocrine disrupting chemicals. J. Mol. Catal. B Enzym..

[62] Lomascolo A., Uzan-Boukhris E., Herpoël-Gimbert I., Sigoillot J.C., Lesage-Meessen L. (2011). Peculiarities of *Pycnoporus* species for applications in biotechnology. Appl. Microbiol. Biotechnol..

[63] Lu L., Zhao M., Zhang B.B., Yu S.Y., Bian X.J., Wang W., Wang Y. (2007). Purification and characterization of laccase from *Pycnoporus sanguineus* and decolorization of an anthraquinone dye by the enzyme. Appl. Microbiol. Biotechnol..

[64] Singh D., Gupta N. (2020). Microbial laccase: A robust enzyme and its industrial applications. Biologia.

[65] Rivera-Hoyos C.M., Morales-Álvarez E.D., Poutou-Piñales R.A., Pedroza-Rodríguez A.M., RodrÍguez-Vázquez R., Delgado-Boada J.M. (2013). Fungal laccases. Fungal Biol. Rev..

[66] Kumar A., Sharma K.K., Kumar P., Ramchiary N. (2015). Laccase isozymes from *Ganoderma lucidum* MDU-7: Isolation, characterization, catalytic properties and differential role during oxidative stress. J. Mol. Catal. B Enzym..

[67] Moldes D., Lorenzo M., Sanromán M.A. (2004). Different proportions of laccase isoenzymes produced by submerged cultures of *Trametes versicolor* grown on lignocellulosic wastes. Biotechnol. Lett..

[68] Janusz G., Pawlik A., Świderska-Burek U., Polak J., Sulej J., Jarosz-Wilkołazka A., Paszczyński A. (2020). Laccase properties, physiological functions, and evolution. Int. J. Mol. Sci..

[69] Bertrand B., Martínez-Morales F., Tinoco-Valencia R., Rojas S., Acosta-Urdapilleta L., Trejo-Hernández M.R. (2015). Biochemical and molecular characterization of laccase isoforms produced by the white-rot fungus *Trametes versicolor* under submerged culture conditions. J. Mol. Catal. B Enzym..

[70] Ayodeji F.D., Shava B., Iqbal H.M.N., Ashraf S.S., Cui J., Franco M., Bilal M. (2022). biocatalytic versatilities and biotechnological prospects of laccase for a sustainable industry. Catal. Lett..

[71] Zhou Z., Li R., Ng T.B., Lai Y., Yang J., Ye X. (2020). A new laccase of Lac 2 from the white rot fungus *Cerrena unicolor* 6884 and Lac 2-Mediated degradation of aflatoxin B_1_. Toxins.

[72] Zeinvand-Lorestani H., Sabzevari O., Setayesh N., Amini M., Nili-Ahmadabadi A., Faramarzi M.A. (2015). Comparative study of in vitro prooxidative properties and genotoxicity induced by aflatoxin B_1_ and its laccase-mediated detoxification products. Chemosphere.

[73] Scarpari M., Bello C., Pietricola C., Zaccaria M., Bertocchi L., Angelucci A., Ricciardi M.R., Scala V., Parroni A., Fabbri A.A. (2014). Aflatoxin control in maize by *Trametes versicolor*. Toxins.

[74] Alberts J.F., Gelderblom W.C.A., Botha A., van Zyl W.H. (2009). Degradation of aflatoxin B_1_ by fungal laccase enzymes. Int. J. Food Microbiol..

[75] Banu I., Lupu A. (2013). Degradation of zearalenone by laccase enzyme. Sci. Study Res. Chem. Chem. Eng. Biotechnol. Food Ind..

[76] Cabral Silva A.C., Venâncio A. (2021). Application of laccases for mycotoxin decontamination. World Mycotoxin J..

[77] Alberts J., Schatzmayr G., Moll W.D., Davids I., Rheeder J., Burger H.M., Shephard G., Gelderblom W. (2019). Detoxification of the fumonisin mycotoxins in maize: An enzymatic approach. Toxins.

[78] Chang K.L., Teng T.C., Fu C.K., Liu C.H. (2019). Improving biodegradation of Bisphenol A by immobilization and inducer. Process. Saf. Environ. Prot..

[79] Li Z., Zhu Q., Liu Z., Sha L., Chen Z. (2022). Improved performance of immobilized laccase for catalytic degradation of synthetic dyes using redox mediators. New J. Chem..

[80] Hu R., Wu S., Li B., Tan J., Yan J., Wang Y., Tang Z., Liu M., Fu C., Zhang H. (2022). Dietary ferulic acid and vanillic acid on inflammation, gut barrier function and growth performance in lipopolysaccharide-challenged piglets. Anim. Nutr..

[81] Singh B., Kumar A., Singh H., Kaur S., Arora S., Singh B. (2022). Protective effect of vanillic acid against diabetes and diabetic nephropathy by attenuating oxidative stress and upregulation of NF-ΚB, TNF-α and COX-2 proteins in rats. Phytother. Res..

[82] Park J., Cho S.Y., Kang J., Park W.Y., Lee S., Jung Y., Kang M.W., Kwak H.J., Um J.Y. (2020). Vanillic acid improves comorbidity of cancer and obesity through STAT3 regulation in high-fat-diet-induced obese and B16BL6 melanoma-injected mice. Biomolecules.

[83] Sharma N., Tiwari N., Vyas M., Khurana N., Muthuraman A., Utreja P. (2020). An overview of therapeutic effects of vanillic acid. Plant Arch..

[84] Zucca P., Rescigno A., Olianas A., MacCioni S., Sollai F.A., Sanjust E. (2011). Induction, purification, and characterization of a laccase isozyme from *Pleurotus sajor-caju* and the potential in decolorization of textile dyes. J. Mol. Catal. B Enzym..

[85] Hernández C.A., Sandoval N., Mallerman J., García-Pérez J.A., Farnet A.M., Perraud-Gaime I., Alarcón E. (2015). Ethanol induction of laccase depends on nitrogen conditions of *Pycnoporus sanguineus*. Electron. J. Biotechnol..

[86] Lomascolo A., Record E., Herpoël-Gimbert I., Delattre M., Robert J.L., Georis J., Dauvrin T., Sigoillot J.C., Asther M. (2003). Overproduction of laccase by a monokaryotic strain of *Pycnoporus cinnabarinus* using ethanol as inducer. J. Appl. Microbiol..

[87] Lee I.Y., Jung K.H., Lee C.H., Park Y.H. (1999). Enhanced production of laccase in *Trametes vesicolor* by the addition of ethanol. Biotechnol. Lett..

[88] Ahmed P.M., Pajot H.F., Fernández P.M., Udayanga D., Bhatt P., Manamgoda D., Saez J.M. (2022). Production of laccases from agricultural wastes: Strain isolation and selection, enzymatic profiling, and lab-scale production. Mycorremediation Protocols.

[89] Kumar R., Kaur J., Jain S., Kumar A. (2016). Optimization of laccase production from *Aspergillus flavus* by design of experiment technique: Partial purification and characterization. J. Genet. Eng. Biotechnol..

[90] Pointing S.B. (1999). Qualitative methods for the determination of lignocellulolytic enzyme production by tropical fungi. Fungal Divers..

[91] Baltierra-Trejo E., Márquez-Benavides L., Sánchez-Yáñez J.M. (2015). Inconsistencies and ambiguities in calculating enzyme activity: The case of laccase. J. Microbiol. Methods.

[92] Bradford M.M. (1976). A rapid and sensitive method for the quantitation of microgram quantities of protein utilizing the principle of protein-dye binding. Anal. Biochem..

[93] Laemmli U.K. (1970). Cleavage of structural proteins during the assembly of the head of bacteriophage T4. Nature.

[94] Arndt C., Koristka S., Bartsch H., Bachmann M., Kurien B.T., Hal Scofield R. (2012). Native Polyacrylamide Gels. Protein Electrophoresis. Methods in Molecular Biology.

[95] Chiotta M.L., Susca A., Stea G., Mulè G., Perrone G., Logrieco A., Chulze S.N. (2011). Phylogenetic characterization and ochratoxin a—Fumonisin profile of black *Aspergillus* isolated from grapes in argentina. Int. J. Food Microbiol..

[96] White T.J., Bruns T., Lee S., Taylor J., Innis M.A., Gelfand D.H., Sninsky J.J., White T.J. (1990). Amplification and direct sequencing of fungal ribosomal RNA genes for phylogenetics. PCR Protocols: A Guide to Methods and Applications.

[97] O’donnell K., Taylor J.W., Reynolds D.R. (1993). Fusarium and its near relatives. The Fungal Holomorph: Mitotic, Meiotic and Pleomorphic Speciation in Fungal Systematics.

[98] Hall T.A. (1999). BioEdit: A user-friendly biological sequence alignment editor and analysis program for Windows 95/98/NT. Nucleic Acids Symp. Ser..

[99] Cendoya E., Chiotta M.L., Zachetti V., Chulze S.N., Ramirez M.L. (2018). Fumonisins and fumonisin-producing *Fusarium* occurrence in wheat and wheat by products: A review. J. Cereal Sci..

[100] Dzuman Z., Zachariasova M., Veprikova Z., Godula M., Hajslova J. (2015). Multi-Analyte High Performance Liquid Chromatography Coupled to High Resolution Tandem Mass Spectrometry Method for control of pesticide residues, mycotoxins, and pyrrolizidine alkaloids. Anal. Chim. Acta.

[101] Di Rienzo J.A., Casanoves F., Balzarini M.G., Gonzalez L., Tablada M., Robledo C.W. (2016). InfoStat, Versión 2018.

